# Conventional Type 1 Dendritic Cells (cDC1) in Human Kidney Diseases: Clinico-Pathological Correlations

**DOI:** 10.3389/fimmu.2021.635212

**Published:** 2021-05-12

**Authors:** Titi Chen, Qi Cao, Ruifeng Wang, Guoping Zheng, Farhana Azmi, Jeffery Wang, Vincent W. Lee, Yuan Min Wang, Hong Yu, Manish Patel, Chow Heok P’ng, Stephen I. Alexander, Natasha M. Rogers, Yiping Wang, David C. H. Harris

**Affiliations:** ^1^ School of Medicine, The University of Sydney, Camperdown, NSW, Australia; ^2^ Centre for Transplant and Renal Research, The Westmead Institute for Medical Research, Westmead, NSW, Australia; ^3^ Department of Renal Medicine, Westmead Hospital, Westmead, NSW, Australia; ^4^ Department of Anatomical Pathology, Westmead Hospital, Westmead, NSW, Australia; ^5^ Department of Urology, Westmead Hospital, Westmead, NSW, Australia; ^6^ Centre for Kidney Research, Children’s Hospital at Westmead, Sydney, NSW, Australia

**Keywords:** dendritic cells, CD141^+^ DCs, conventional DCs, crescent, interstitial fibrosis, glomerulonephritis, acute tubular necrosis

## Abstract

**Background:**

cDC1 is a subset of conventional DCs, whose most recognized function is cross-presentation to CD8^+^ T cells. We conducted this study to investigate the number and location of cDC1s in various human kidney diseases as well as their correlation with clinico-pathological features and CD8^+^ T cells.

**Methods:**

We analyzed 135 kidney biopsies samples. Kidney diseases included: acute tubular necrosis (ATN), acute interstitial nephritis (AIN), proliferative glomerulonephritis (GN) (IgA nephropathy, lupus nephritis, pauci-immune GN, anti-GBM disease), non-proliferative GN (minimal change disease, membranous nephropathy) and diabetic nephropathy. Indirect immunofluorescence staining was used to quantify cDC1s, CD1c^+^ DCs, and CD8^+^ T cells.

**Results:**

cDC1s were rarely present in normal kidneys. Their number increased significantly in ATN and proliferative GN, proportionally much more than CD1c^+^ DCs. cDC1s were mainly found in the interstitium, except in lupus nephritis, pauci-immune GN and anti-GBM disease, where they were prominent in glomeruli and peri-glomerular regions. The number of cDC1s correlated with disease severity in ATN, number of crescents in pauci-immune GN, interstitial fibrosis in IgA nephropathy and lupus nephritis, as well as prognosis in IgA nephropathy. The number of CD8^+^ T cells also increased significantly in these conditions and cDC1 number correlated with CD8^+^ T cell number in lupus nephritis and pauci-immune GN, with many of them closely co-localized.

**Conclusions:**

cDC1 number correlated with various clinic-pathological features and prognosis reflecting a possible role in these conditions. Their association with CD8^+^ T cells suggests a combined mechanism in keeping with the results in animal models.

## Introduction

Dendritic cells (DCs) are the central orchestrators of effective immunity. These cells are heterogeneous and can be divided into distinct subsets based on their phenotype and function. Dendritic cells can be broadly categorized into plasmacytoid DCs (pDC) and conventional DCs (cDC). cDC consists of two major subsets: cDC1 (CD141^+^ DCs in humans and CD103^+^ or CD8α^+^ DCs in rodents) and cDC2 (CD1c^+^ DCs in human and CD11b^+^ DCs in rodents) (1). cDC1 were discovered first in mouse and then in humans and are characterized by their superior ability to phagocytose necrotic cells through damage-associated molecular pattern (DAMP) receptor Clec9A and to cross-present to CD8^+^ T cells. In contrast, cDC2s are effective CD4^+^ T cell activators but inferior at CD8^+^ T cell activation (2).

DCs have been studied in human kidney disease and their number was found to be increased in glomerulonephritis (GN) (3, 4). After the discovery of cDC1, accumulating animal studies have shown that they play a pivotal role in kidney diseases, such as in adriamycin nephropathy and crescentic GN, through interaction with T cells (5–8). However, such studies are lacking in human kidney disease, with only one study demonstrating increased numbers of cDCs in GN (9). We conducted this study to provide analysis of the key cDC subsets, cDC1 and cDC2, in a wide range of human kidney diseases including non-glomerular diseases [acute tubular necrosis (ATN), acute interstitial nephritis (AIN)], proliferative GN (IgA nephropathy, lupus nephritis, pauci-immune GN, anti-GBM disease), non-proliferative GN (minimal change disease (MCD), membranous nephropathy) and diabetic nephropathy. We found cDC1s to be significantly correlated with pathological features including severity of ATN, crescent formation in pauci-immune GN and interstitial fibrosis in immune-mediated GN. In addition, consistent with their specialized ability to activate CD8^+^ T cells in animal models, we also demonstrated their correlation with CD8^+^ T cells in these samples. These findings provide an impetus to explore new therapeutic targets that manipulate these cells for treatment of kidney diseases.

## Material and Methods

### Patients and Tissue Samples

The study was conducted in accordance with principles of the Declaration of Helsinki and was approved by the Human Research Ethics Committee of the Western Sydney Local Health District. Informed patient consent was obtained.

We analyzed 176 frozen diagnostic kidney biopsy samples taken from non-pregnant adult patients (>18 years old) between 1^st^ June 2016 to 30^th^ June 2017 in Westmead Hospital, Sydney, Australia. Kidney tissues were snap frozen in O.C.T. compound (Tissue-Tek Sakura, USA) and stored at -80°C. Forty-one (41) samples had poor tissue quality and were excluded from the study. Baseline patient data including age, gender, estimated glomerular filtration rate (eGFR) and degree of proteinuria were collected at the time of kidney biopsy. eGFR was calculated using the CKD-EPI formula. The diagnosis was made by a renal pathologist, based on light, immunofluorescence (IF) and electron microscopy as well as clinical history.

A variety of diseases were analyzed including non-glomerular diseases (ATN, AIN), proliferative GN (IgA nephropathy, lupus nephritis, pauci-immune GN, anti-GBM disease), non-proliferative GN (MCD, membranous nephropathy) and diabetic nephropathy. For ATN, we only included non-septic cases as septic ATN has a different pathophysiology. ATN was further categorized into mild-moderate disease (defined as <50% cortical tubules showing injury) and severe disease (>=50% cortical tubules showing injury). IgA nephropathy was classified according to the Oxford MEST score (M mesangial hypercellularity, E endocapillary hypercellularity, S segmental glomerulosclerosis, T tubular atrophy/interstitial fibrosis). We also analyzed the correlation between cDC1 number and prognosis (defined *a priori* as >20% reduction in eGFR on or before 31^st^ December 2019) in IgA nephropathy by dividing patients into 2 groups according to cDC1 number with cut-off point at upper quartile (>=15). Lupus nephritis was further classified into 2 groups according to the level of interstitial fibrosis (<25%, >=25% cortical interstitial involvement). Pauci-immune GN was classified into 2 groups according to the percentage of glomeruli with crescents (<40%, >=40%).

As normal kidney controls, we used 5 normal renal cortices from nephrectomy samples as well as 2 donor kidneys not suitable for transplant. For tumor nephrectomies, samples were taken from the pole opposite to the tumor and at least 5 cm from the tumor margin. These tissues had normal macroscopic appearance. Microscopically, none of these kidney samples had evidence of significant glomerular or tubulointerstitial inflammation or injury. We used normal adult donor splenic tissue as positive control for testing antibodies.

### Immunofluorescence Staining

Serial cryostat sections were cut at 5μm and placed onto Superfrost Ultra Plus glass slides (Thermo Scientific, USA). Slides were stored at -80°C. Tissue sections were fixed with 100% methanol at -20°C for 10 minutes and then air-dried. Indirect immunofluorescence staining was performed using the following method: tissue sections were washed in DPBS (Lonza, USA) and blocked with 2% Bovine Serum Albumin (Sigma-Aldrich, USA) for 15 minutes; then they were stained with primary antibody at 4°C overnight followed by secondary antibodies for 40 minutes at room temperature. **Table 1** is a list of primary and secondary antibodies used and their dilutions. Nuclei were stained with DAPI (1:250,000, ThermoFisher, USA) before samples were mounted on coverslips with fluorescence mounting medium (Dako, USA). Non-specific staining and cross reactivity between different primary and secondary antibodies were checked and excluded.

**Table 1:** Primary and secondary antibodies used in the study.

Primary antibody	Host	Clone	Company	Dilution
Clec9A	Mouse anti-Human	8F9	Miltenyi Biotec	1:25
CD1c	Mouse anti-Human	L161	Biolegend	1:100
HLA-DRB1	Rabbit anti-Human	EPR1126	Abcam	1:100
CD11c	Rabbit anti-human	EP1347Y	Abcam	1:100
CD8	Rabbit anti-Human	SP16	Invitrogen	1:100
**Secondary antibody**				
Alexa Fluor 647	Donkey anti-Rabbit		Invitrogen	1:1,600
Alexa Fluor 546	Goat anti-Mouse		Invitrogen	1:1,600
Alexa Fluor 546	Goat anti-Rabbit		Invitrogen	1:1,600

cDC1s were identified by staining for marker Clec9A. In humans, Clec9A expression is highly restricted to cDC1s in blood and tissues (10, 11). To confirm this in the kidneys, we performed double staining of Clec9A and HLA-DRB1 as well as Clec9A and CD11c in selected normal and diseased conditions. Most, if not all Clec9A overlapped with HLA-DRB1 (**Supplementary Figure 1**) and CD11c (**Supplementary Figure 2**), indicating that Clec9A accurately identified the cDC1 subset. cDC2 were identified by staining for marker CD1c and double staining of CD1c with HLA-DRB1 and CD11c was also performed to ensure accuracy. CD8^+^ T cells were identified by staining for marker CD8.

### Quantification of DC Numbers and Statistical Analysis

IF images were acquired with a confocal microscope (Olympus FV 1000 confocal laser scanning microscope). Four randomly selected pictures were taken for each sample under 20X magnification and the average number of positive cells in these 4 pictures was used in the analysis. Labeled cells were counted by an investigator twice in a blinded fashion and the mean value was used. When calculating cDC2/cDC1 ratio, we used the mean number of cDC2 and cDC1 for each sample. When cDC1 number was 0, we used 1 instead of 0 in calculating cDC1/cDC1 ratio as it would otherwise be infinite. The number of cells was expressed per high power field. In quantifying the number of cells in the intra-glomerular region, 4 randomly selected glomeruli were taken from each sample and the average number of cells inside these 4 glomeruli was calculated. Image J manual cell counting and marking tool was used to record positive cells.

Statistical analysis was performed using SPSS version 26. All analyses were 2 tailed and P<0.05 was considered statistically significant. When there was multiple comparisons to the control group (5), Bonferroni adjustment was made and P<0.01 was considered statistically significant. Continuous variables were presented as median with interquartile range (IQR). The distributions between groups were compared using Mann-Whitney U-test or Kruskal Wallis tests as appropriate. The strength of association was quantified using Spearman rank correlation. Kaplan-Meier time-to-event curves with log-rank test were used for outcome analysis.

## Results

### Patient’s Baseline Characteristics

The baseline characteristics of patients in control and disease cohorts are summarized in **Table 2**. A total of 135 patients were included in the study. A wide range of kidney diseases were analyzed including non-glomerular diseases [ATN (22), AIN(10)], proliferative GN [IgA nephropathy (44), lupus nephritis (12), pauci-immune GN (12), anti-GBM disease (4)], non-proliferative GN [MCD (5), membranous nephropathy (5)] and diabetic nephropathy (21). There were more females in the lupus nephritis group and their age tended to be younger compared with other kidney diseases, which is consistent with literature (12). Patients with pauci-immune GN and anti-GBM disease had the lowest eGFR. The proteinuria level was the highest in MCD and membranous nephropathy.

**Table 2:** Baseline characteristics.

	Patient number	Gender (Male)	Age (years)	eGFR (ml/min/1.73^2^)	Proteinuria (g/24 hours)
Control	7	4	54 (40-64)	86 (79-90)	N/A
**Non glomerular disease**					
ATN	22	16	59 (43-69)	22 (18-40)	0.6 (0.2-0.8)
AIN	10	7	48 (26-72)	28 (21-36)	0.4 (0.3-0.7)
**Proliferative GN**					
IgA	44	31	47 (37-63)	43 (31-73)	2.2 (0.9-4.0)
Lupus nephritis	12	4	29 (26-37)	71 (30-90)	3.2 (0.8-5.1)
Pauci-immune	12	9	67 (61-77)	14 (8-30)	4.1 (1.4-5.0)
Anti-GBM	4	3	57 (33-66)	13 (4-23)	N/A
**Non Proliferative GN**					
Minimal change disease	5	1	50 (48-62)	82 (63-90)	10.2 (5.0-14.5)
Membranous nephropathy	5	2	51 (38-78)	33 (14-77)	4.3 (2.9-10.8)
**Other non immune mediated glomerular disease**					
Diabetic nephropathy	21	11	68 (47-74)	33 (23-55)	3.3 (2.0-6.5)

Data are expressed as median (IQR). N/A indicates data not available.

### Number and Location of DCs in Control and Disease

**Figure 1:**
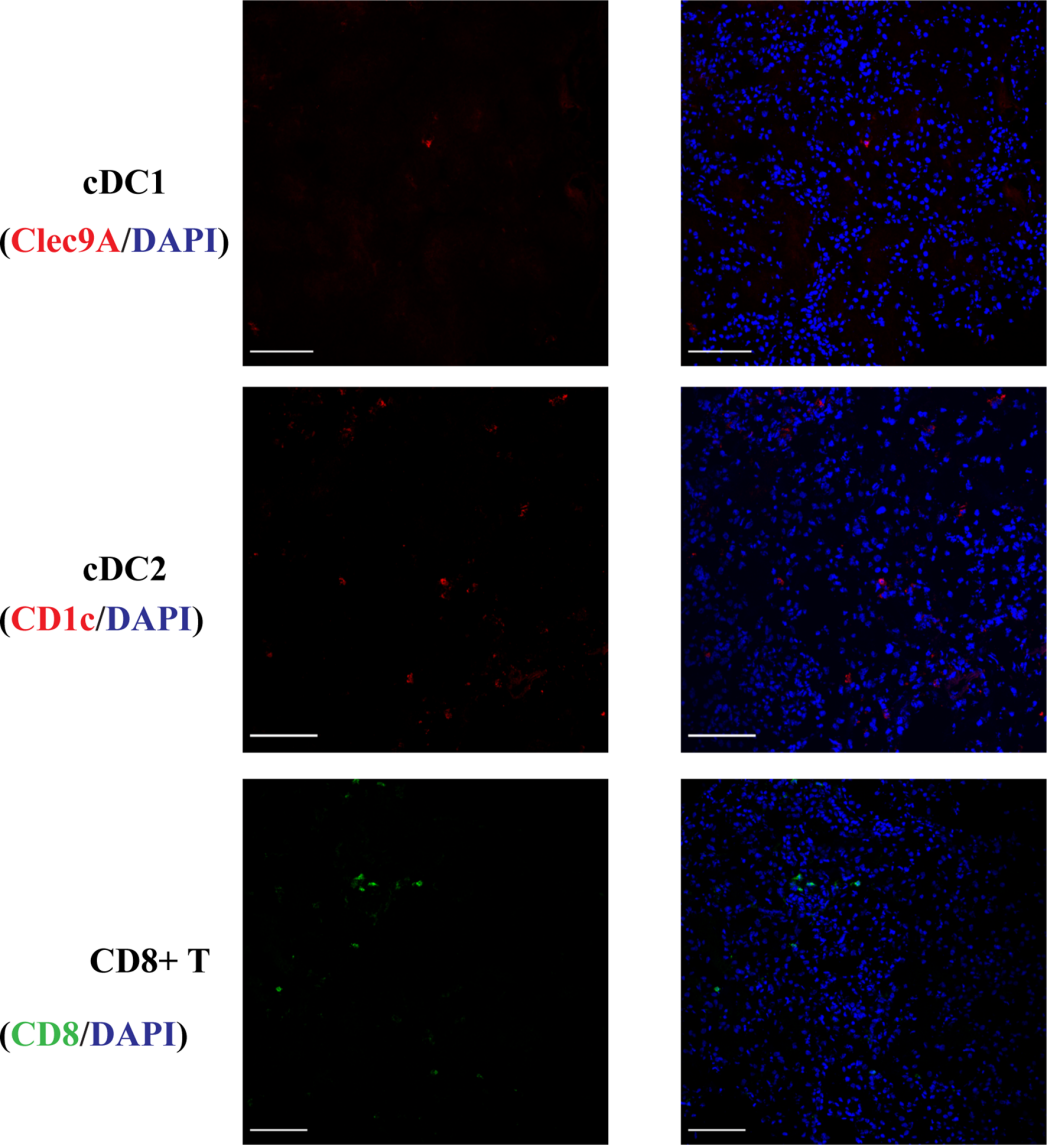
Normal kidney cDC1, cDC2 and CD8^+^ T cells. DCs were rarely present in normal kidneys and cDC2 numbers were approximately 7 times the number of cDC1. (Bar = 100 μm).

cDC1s were rarely present in normal kidneys (**Figure 1**) and cDC2 numbers were approximately 7 times the number of cDC1.

**Figure 2:**
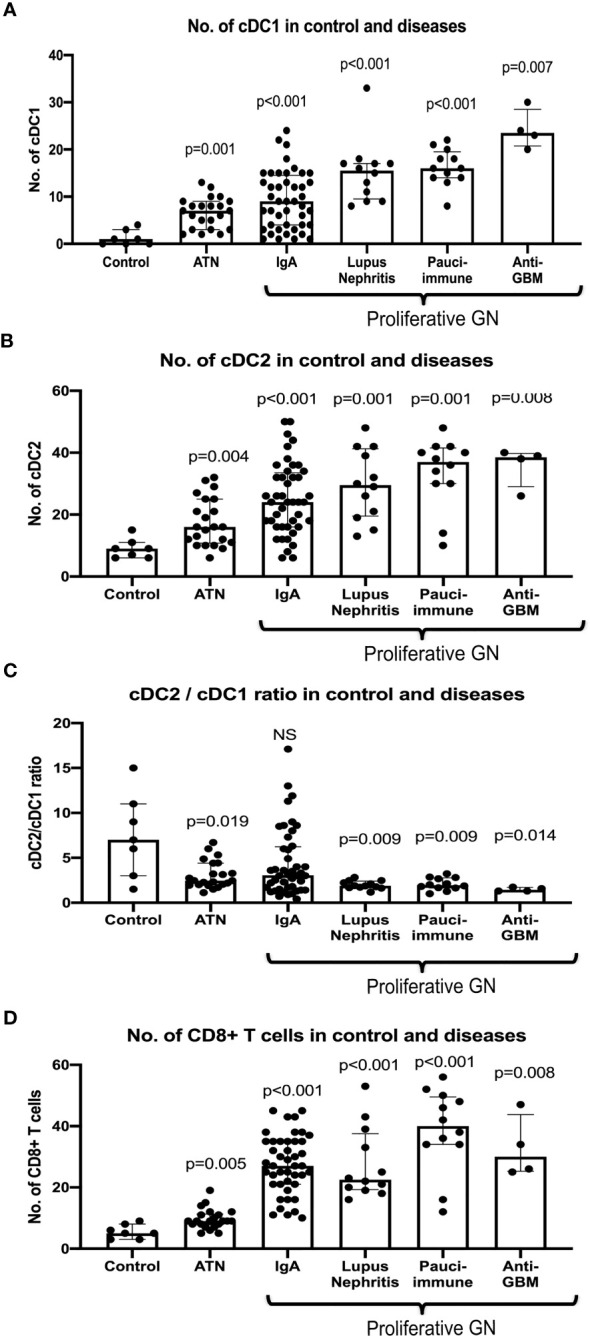
Number of cDC1 and cDC2, cDC2/cDC1 ratio and CD8^+^ T cell in control and selected diseases. P value is calculated for each disease *versus* control. Both cDC1 and cDC2 increased significantly in ATN, IgA, lupus nephritis, pauci-immune GN and anti-GBM disease **(A, B)** with cDC1 increased proportionally more than cDC2 in ATN, lupus nephritis, pauci-immune GN and anti-GBM disease **(C)**. CD8^+^ T cells were increased significantly in ATN, IgA, lupus nephritis, pauci-immune GN and anti-GBM disease **(D)**.

The number of cDC1 increased significantly in ATN and proliferative GN (**Figure 2A**), while their number remained unchanged compared to control in AIN, membranous nephropathy, MCD and diabetic nephropathy (**Supplementary Figure 3**). The number of cDC2 also increased significantly in ATN and proliferative GN (**Figure 2B**). There was a reduction in the cDC2/cDC1 ratio indicating cDC1 increased proportionally more than cDC2 (**Figure 2C**).

**Figure 3:**
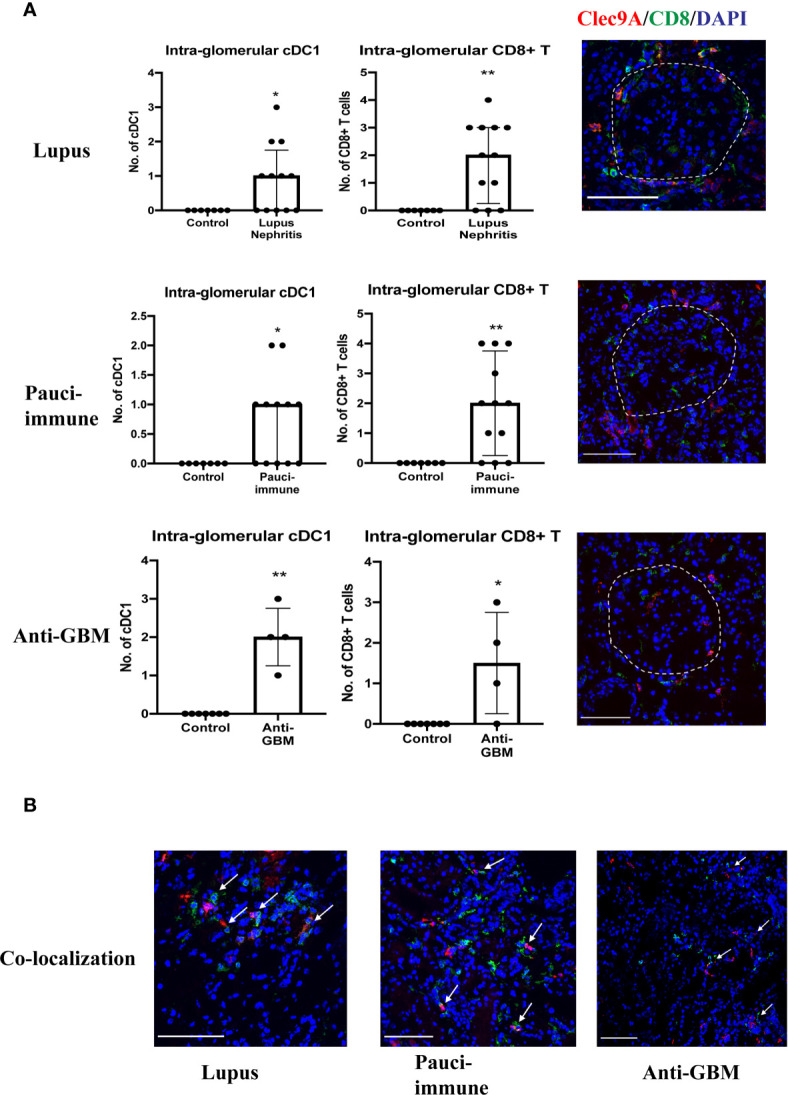
**(A)** cDC1 and CD8^+^ T cells in intra-glomerular regions. **(B)** cDC1 co-localization with CD8^+^ T cells. (Bar = 100 μm). * P < 0.05, ** P < 0.01.

Most cDC1s were located in the interstitium, except in lupus nephritis, pauci-immune GN and anti-GBM disease where they were also found in peri-glomerular and intra-glomerular regions (**Figure 3A**). In addition, we also found a significant number of CD8^+^ T cells in peri-glomerular and intra-glomerular regions (**Figure 3A**), and many of them co-localized with cDC1s (**Figure 3B**). On the other hand, cDC2s were rarely found in intra-glomerular regions and there was minimal co-localization with CD8^+^ T cells.

### Association Between cDC1 With Clinical-Pathological Features and CD8^+^ T Cells

We analyzed the correlation between cDC1 and clinicopathological features as well as CD8^+^ T cells.

**Figure 4:**
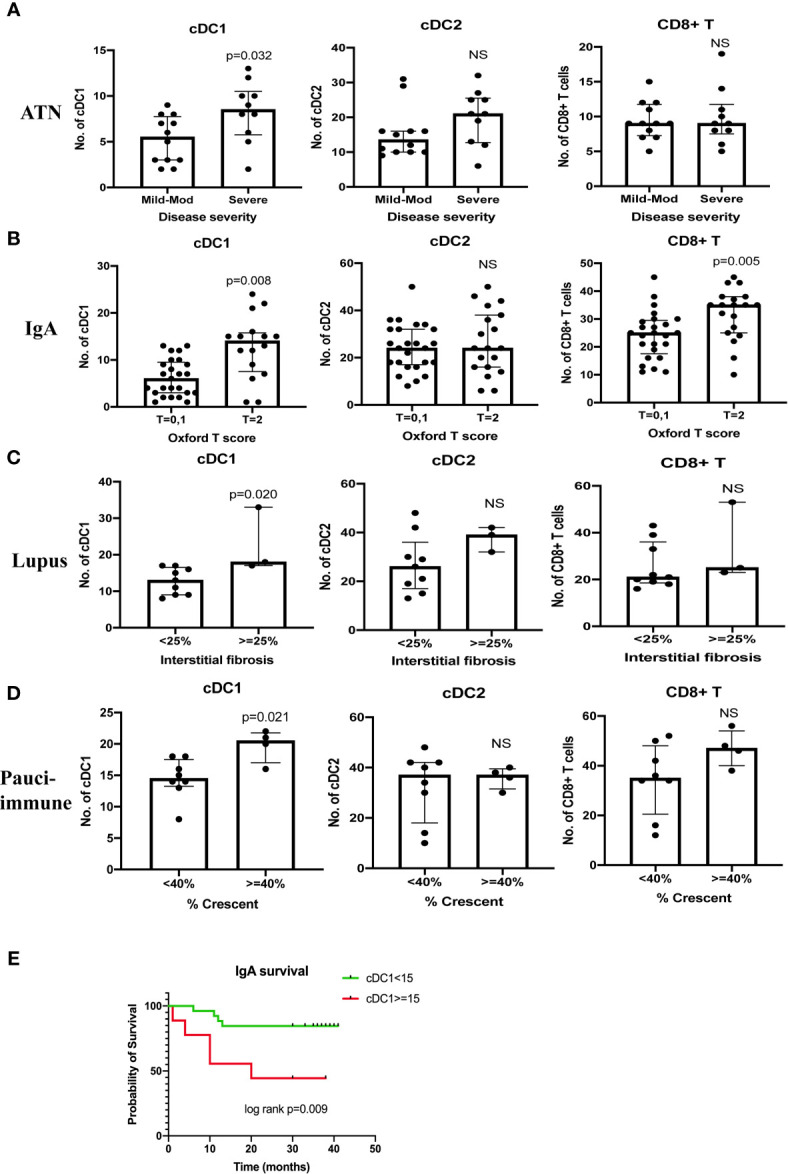
Correlation between cDC1, cDC2 and CD8^+^ T cell number and disease severity **(A–D)**. Kaplan Meier curve of IgA nephropathy survival **(E)**. T score 0 refers to the percentage of area showing tubular atrophy/interstitial fibrosis < = 25%, T score 1 refers to the percentage of area showing tubular atrophy/interstitial fibrosis 26 - 50%, T score 2 refers to the percentage of area showing tubular atrophy/interstitial fibrosis > 50%. Survival was defined as > 20% reduction in eGFR on or before 31^st^ December 2019. NS, not significant.

There were 22 cases of ATN (mild - mod disease n=12, severe disease n=10). More severe disease was associated with a higher number of cDC1 (p=0.032), but not cDC2 (**Figure 4A**). cDC1 increased proportionally more than cDC2 (cDC2/cDC1 ratio 2.5, p=0.019). The number of CD8^+^ T cells also increased significantly in ATN (p=0.005) (**Table 3**, **Figure 2D**). The number of cDC1 did not correlate with CD8^+^ T cell number in ATN.

**Table 3:** CD8+ T cell number and correlation coefficient between cDC1 and CD8+T cells numbers in control and diseased kidneys.

	CD8^+^ T cell number		cDC1 and CD8^+^ T Correlation coefficient
	Median (IQR)	p	
Control	5 (3-8)	Reference	NS
ATN	9 (7-11)	0.005	NS
IgA	25 (21-34)	<0.001	NS
Lupus nephritis	22 (19-37)	<0.001	0.614 (p=0.034)
Pauci-immune	40 (34-49)	<0.001	0.644 (p=0.024)
Anti-GBM	30 (25-43)	0.008	NS

Data are expressed as median (IQR). NS indicates not significant. Statistical significance was assessed between control and disease.

Forty-four cases of IgA nephropathy were analyzed. Using the Oxford classification MEST score (M mesangial hypercellularity, E endocapillary hypercellularity, S segmental glomerulosclerosis, T tubular atrophy/interstitial fibrosis), a higher number of cDC1 was associated with a higher T score (p=0.008), but not MES scores (**Figure 4B**). There were 7 cases with crescents and the number of cCD1 was not associated with the number of crescents. The number of kidney CD8^+^ T cells was significantly higher in IgA nephropathy than control (p<0.001) (**Table 3**, **Figure 2D**). There was no correlation between cDC1 and CD8^+^ T cell number. Thirty-five (35) patients had follow up data on or before 31^st^ December 2019, of whom 9 experienced > 20% reduction in eGFR. Dividing patients into 2 groups according to cDC1 number with cut-off point at upper quartile (>=15), the higher cDC1 number group was associated with worse outcome (**Figure 4E**).

There were 12 cases of lupus nephritis. As in IgA nephropathy, higher cDC1 number was associated with more severe fibrosis (p=0.020) (**Figure 4C**). In addition, CD8^+^ T cell number also increased significantly (P<0.001) (**Table 3**, **Figure 2D**) and this correlated with the number of cDC1 cells (r=0.614, p=0.034) (**Table 3**). A significant number of cDC1 and CD8^+^ T cells were found in peri-glomerular as well as intra-glomerular regions (**Figure 3A**). Many cDC1s co-localized with CD8^+^ T cells (**Figure 3B**).

Twelve cases of pauci-immune GN were included in the analysis. A higher number of cDC1 was found in the group with crescents >40% (p*=*0.021*)* (**Figure 4D**). The number of CD8^+^ T cells was also significantly higher than in control kidneys (p<0.001) (**Table 3**, **Figure 2D**). The number of cDC1s correlated with that of CD8^+^ T cells (r=0.644, p=0.024) (**Table 3**). As in lupus nephritis, there was a prominent peri-glomerular cDC1 and CD8^+^ T cell infiltration (**Figure 3A**). There were also intra-glomerular cDC1s and CD8^+^ T cells (**Figure 3A**). Numerous cDC1 co-localized with CD8^+^ T cells (**Figure 3B**).

There were 4 cases of anti-GBM disease. cDC1, cDC2 and CD8^+^ T cell numbers increased significantly compared to control, with cDC1s increased proportionally much more than cDC2s (**Figures 2, 2D**). However, there was no correlation between cDC1 and CD8^+^ T cell number. There were also numerous cDC1 and CD8^+^ T cells in the peri-glomerular region as well as inside the glomerulus (**Figures 3A, B**).

## Discussion

This is the first study providing a detailed analysis of different cDC subsets in healthy and diseased human kidney. This study provides several important findings; first, in non-septic ATN where innate immunity plays a central role, we found higher numbers of cDC1s, which increased proportionally more than cDC2s. cDC1 number also correlated with disease severity indicating they may play a role in this condition, Second, in interstitial fibrosis associated immune-mediated disease (IgA nephropathy and lupus nephritis), we found that cDC1 number correlated with severity of fibrosis, as well as prognosis in IgA nephropathy, while no such correlation was found in non-immune mediated fibrotic disease (diabetic nephropathy). Third, there was a strong correlation between cDC1 number and crescent formation in pauci-immune GN, and cDC1 were present in large numbers in peri-glomerular and intra-glomerular regions indicating their possible role in crescent formation. Fourth, the number of cDC1 correlated with CD8^+^ T cell numbers in lupus nephritis and pauci-immune GN, with numerous cDC1s co-localized with CD8^+^ T cells suggesting their possible interaction. This is keeping with our findings in animal models of kidney disease that show murine homologs of cDC1 cells preferentially activate CD8^+^ T cells. Taken together, these findings suggest that cDC1 play an important role across a range of kidney diseases including ATN, interstitial fibrosis in immune-mediated disease and crescent formation, and that they may potentially act through activation of CD8^+^ T cells.

### cDC1 Number Correlated With Disease Severity in ATN

In non-septic ATN, for the first time, we showed a significantly increased number of DCs, especially cDC1s compared with cDC2s. Importantly, the cDC1 number correlated with disease severity. In addition, CD8^+^ T cell number was also increased. ATN is usually caused by ischemia reperfusion injury (IRI), nephrotoxins or sepsis. Traditionally, IRI and toxins cause sterile inflammation, and innate immunity, where DCs and T cells are less important, was considered to play a dominant role. However, increasing evidence from animal studies has shown both cDC1 and CD8^+^ T cells are important players. Previous studies in animal IRI and cisplatin nephrotoxicity found the total DC and T cell numbers increased (13–17). Our previous studies showed in Adriamycin nephropathy, cDC1 numbers increased significantly (5). In IRI, a subset of activated dendritic cells demonstrate increased capacity to cross present antigen to CD8^+^ T cells (18). In Adriamycin nephropathy, we also found cDC1s elicited a CD8^+^ T cell response leading to injury (5). The lack of a correlation between cDC1 and CD8^+^ T cell in human ATN in contrast to the findings in animal models may reflect different non-immunological pathways of injury in human ATN compared to the IRI in mouse models where cDC1s play a significant role through CD8^+^ T cells. In addition, it has been demonstrated in rodents that cDC1 are recruited into the tissue by chemoattractant XCL1 produced by natural killer cells (19). Therefore, it may be worthwhile to study this further in kidney disease.

### cDC1 Number Correlated With Immune-Mediated Interstitial Fibrosis

The second significant finding in this study is that cDC1 number correlated with the severity of interstitial fibrosis associated with immune-mediated disease (IgA nephropathy and lupus nephritis), but not in non-immune mediated fibrotic disease (diabetic nephropathy), Previous study showed increased numbers of cDC1s and cDC2s in interstitial fibrosis (9). We, for the first time, showed that this is only true in immune-mediated disease and cDC1 number increased proportionally more than cDC2s. In addition, we also demonstrated cDC1 number correlated with prognosis and the number of CD8^+^ T cells also increased significantly in IgA nephropathy. This is supported by animal studies showing DCs directly contribute to fibrosis. For example, DC-derived amphiregulin promoted fibrosis (20). It is also possible that cDC1s contributed to fibrosis through CD8^+^ T cells, which are known to contribute to fibrosis in other organs (21–23). Since interstitial fibrosis is linked to the progression of chronic kidney disease, it is not surprising that we found cDC1 number to be a good prognostic marker in IgA nephropathy. Other studies have shown CD8^+^ T cells correlated with prognosis of IgA nephropathy (24), which may be the result of cross presentation from cDC1s. In lupus nephritis, we demonstrated a correlation between cDC1 number and interstitial fibrosis as well as number of CD8^+^ T cells. Previous studies have also shown an increased kidney cDC1 number in lupus nephritis (25), specially class III and VI lupus nephritis, with a corresponding reduction in their circulating numbers (26). We extended these findings by showing that they also correlated with chronic changes. It is well established that interstitial inflammation, which is comprised of T cells, B cells, dendritic cells and macrophages, has a dominant role in the progression of lupus nephritis (27). cDC1s may contribute to the progression of lupus nephritis in a variety of ways. First, activation of interferon plays a key role in the pathogenesis of lupus nephritis (28–30) and cDC1s are a prominent producer of IFN-λ. (31) Second, cDC1 may contribute to lupus nephritis progression through CD8^+^ T cells (32–37) and our finding of a correlation between cDC1s and CD8^+^ T further supports their possible interaction. The role of CD8^+^ T cells in lupus nephritis has been previously demonstrated. CD8^+^ T cells control autoreactive immunity by release of cytotoxic molecules. CD8^+^ T cells in lupus nephritis were found to have dampened cytotoxic function, which can trigger autoimmunity (38). In addition, these cells can also generate lupus autoantigens (39). There has been abundant evidence that CD8^+^ T cells in both kidney (32, 33) and urine (34–36) correlate with disease activity and histological injury in lupus nephritis. In addition, CD8^+^ T-cell exhaustion was shown to predict a favorable prognosis (37).

### cDC1 Number Correlated With the Number of Crescents in Pauci-Immune GN

In pauci-immune GN, we found cDC1s aggregated in the peri-glomerular and intra-glomerular regions and their number correlated with the number of crescents and CD8^+^ T cells. Previous studies showed that DCs are rarely present inside the glomerulus (3, 4, 9) or only in very small numbers (40). On the other hand, T cells were prominent in interstitium, peri-glomerular and intra-glomerular regions (41–44). We found that both cDC1s and CD8^+^ T cells are prominent in the peri-glomerular and intra-glomerular regions with many of them co-localized. In addition, cDC1 number correlated with crescent and CD8^+^ T cell number. All of these findings suggest a role for cDC1 in crescent formation through interaction with CD8^+^ T cells. The pathogenic role of CD8^+^ T cells in pauci-immune GN and crescent formation has already been demonstrated in animal models (45, 46). Consistent with our findings in humans, in animal crescentic GN, cDC1 and CD8^+^ T cells were found especially in the periglomerular region (47, 48). It has been shown that Bowman’s capsule provides a protected immunological niche by preventing access of DCs and cytotoxic CD8^+^ T cells to Bowman’s space and thereby podocytes (45, 47). However, when Bowman’s capsule was breached, these inflammatory cells gained access and destroyed podocytes resulting in rapidly progressive GN (47).

One limitation of this study is that the IF staining technique allows the use of only a limited number of markers. It would be beneficial to extend our findings using technology such as flow cytometry, which can combine multiple markers to further analyze the phenotype of these DCs and their relevant cytokine and chemokine profiles. However, location information will be lost. Other techniques such as multiplex immunohistochemistry and Nanostring can also be considered in future studies to further examine these cells in kidney disease. In addition, when staining cDC2s using CD1c, HLA-DRB1 and CD11c, there may be a small percentage of B cells that express these markers as well, which has not been ruled out.

## Conclusions

Even though cDC1 comprise a minor subset of DCs under homeostatic conditions, this study demonstrates significant correlation between this cell population and clinico-pathological features in human kidney disease. This reflects their likely importance in disease processes such as ATN, crescent formation in proliferative GN and interstitial fibrosis in immune-mediated GN. In addition, their co-localization and correlation with CD8^+^ T cells may provide an explanation for their mechanism of action, corroborating data from animal models. These findings provide an impetus to explore new therapeutic targets that manipulate these cells for treatment of kidney diseases, as we have done in animal studies (6), and to investigate their use as a prognostic marker. Further studies in both humans and animals are needed to interrogate the role of cDC1s, their mechanism of action and how best to target them therapeutically.

## Data Availability Statement

The original contributions presented in the study are included in the article/**Supplementary Material**. Further inquiries can be directed to the corresponding author.

## Ethics Statement

The studies involving human participants were reviewed and approved by Human Research Ethics Committee of the Western Sydney Local Health District. The patients/participants provided their written informed consent to participate in this study.

## Author Contributions 

YPW, QC, TC, and DH designed the study. QC, TC, RW, JW, and FA carried out experiments; QC, HY, CP, and TC interpreted and analyzed the data. TC made the figures. NR, DH, YMW, VL, SA, and GZ provided intellectual input and expertise; MP collected nephrectomy tissue samples. TC, QC, YPW, and DH drafted and revised the paper. All authors contributed to the article and approved the submitted version.

## Funding

This work was supported by the National Health and Medical Research Council of Australia (grant APP1141330).

## Conflict of Interest

The authors declare that the research was conducted in the absence of any commercial or financial relationships that could be construed as a potential conflict of interest.

## Supplementary Material

Supplementary Figure 1 Double staining of Clec9A with HLA-DRB1.

Supplementary Figure 2 Double staining of Clec9A with CD11c.

Supplementary Figure 3 Number of cDC1 in AIN, membranous, MCD and diabetic nephropathy, which didn’t show an increase in these conditions.
