# Supplementary figures and images for: Conventional Type 1 Dendritic Cells (cDC1) in Human Kidney Diseases: Clinico-Pathological Correlations
Source: Front Immunol. 2021 May 12;12:635212. doi: 10.3389/fimmu.2021.635212 (PMC8149958; doi:10.3389/fimmu.2021.635212)

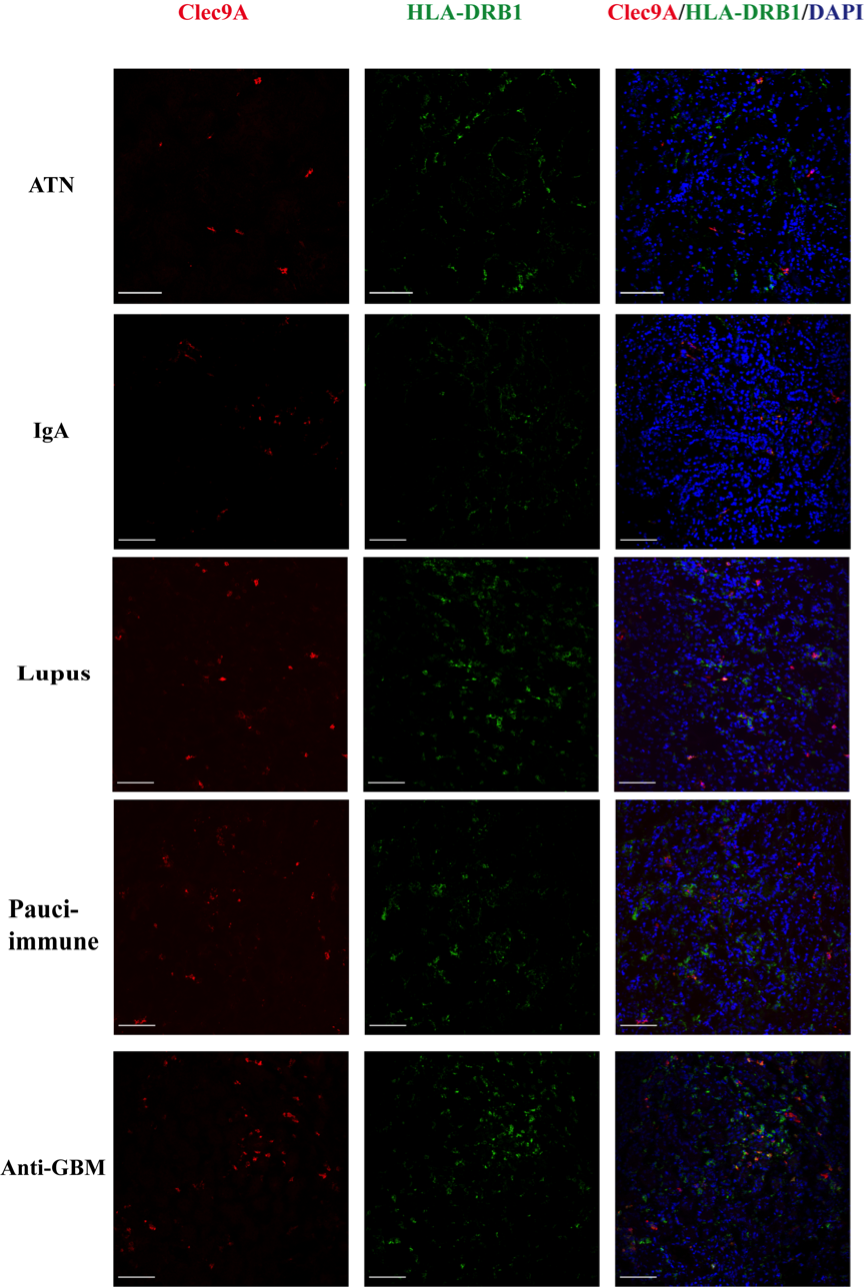

Supplement: Supplementary Figure 1 — Double staining of Clec9A with HLA-DRB1. [file Image_1.tif]

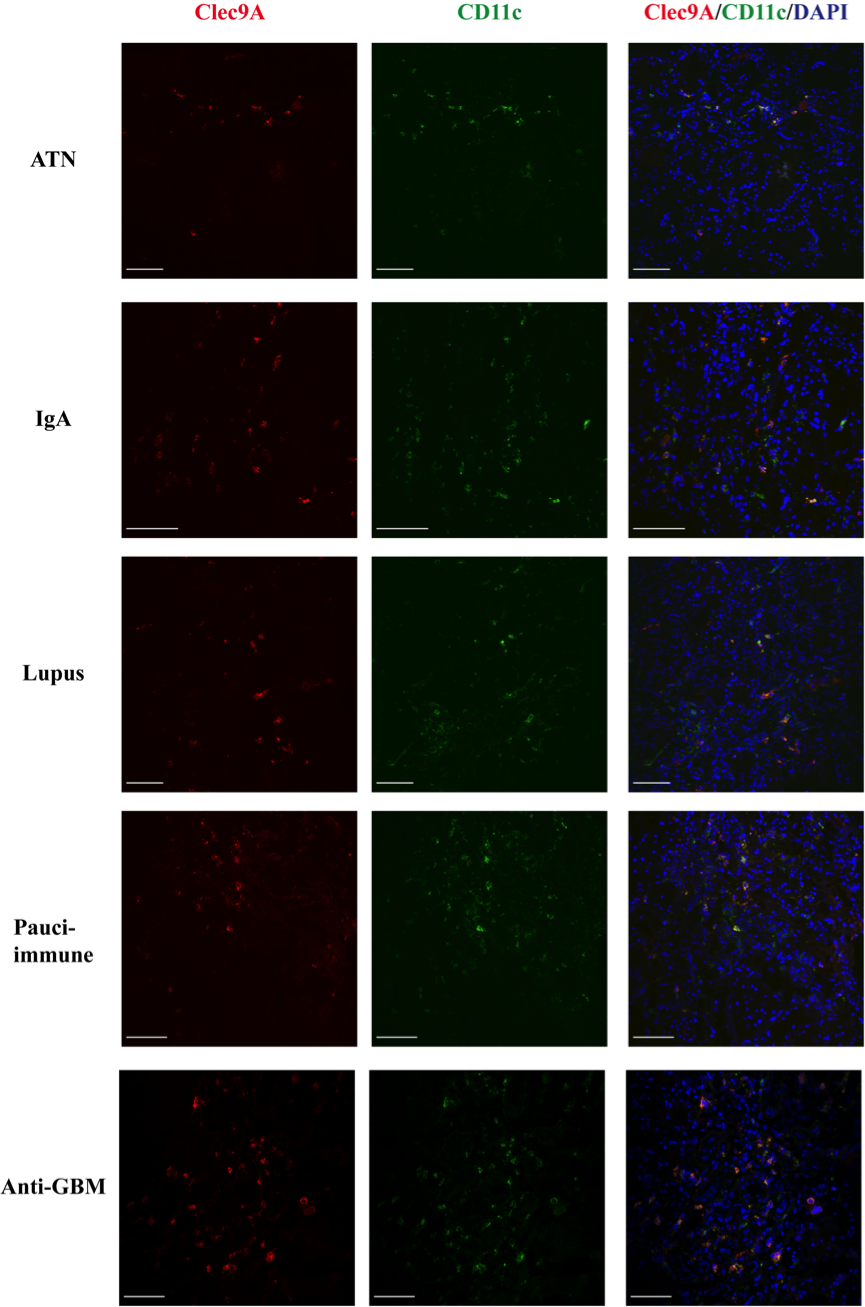

Supplement: Supplementary Figure 2 — Double staining of Clec9A with CD11c. [file Image_2.tif]

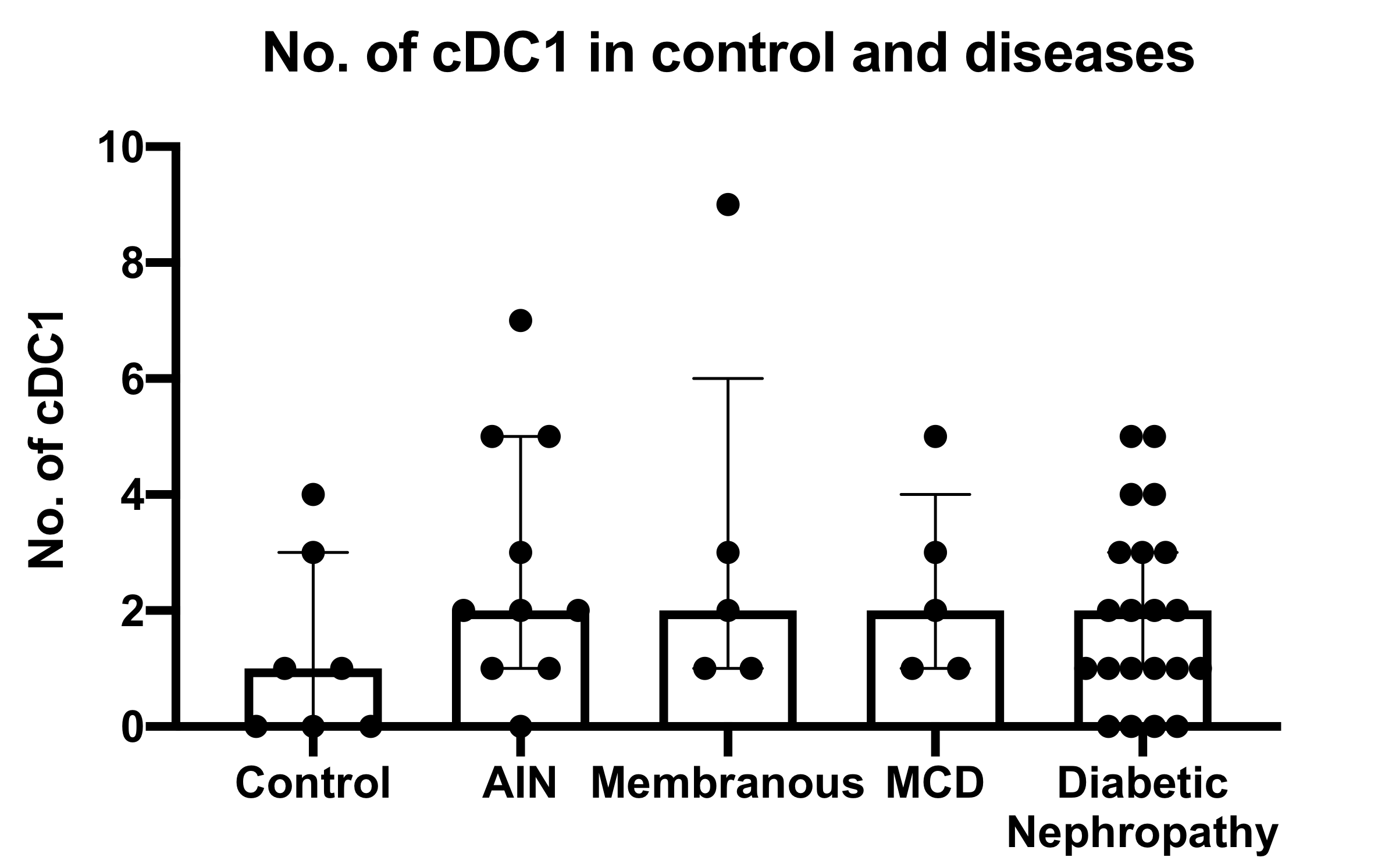

Supplement: Supplementary Figure 3 — Number of cDC1 in AIN, membranous, MCD and diabetic nephropathy, which didn’t show an increase in these conditions. [file Image_3.tiff]
